# Effect of age, gestation and lactation on faecal IgA and calprotectin concentrations in dogs*

**DOI:** 10.1017/jns.2014.44

**Published:** 2014-09-30

**Authors:** Aurélien Grellet, Hanna Mila, Romy M. Heilmann, Alexandre Feugier, Niels Gruetzner, Jan S. Suchodolski, Jorg M. Steiner, Sylvie Chastant-Maillard

**Affiliations:** 1 Royal Canin Research Center, 650 avenue de la Petite Camargue, Aimargues 30 470, France; 2 Unité Toulousaine d'Elevage et Reproduction (UTER), UMR INRA/ENVT 1225 IHAP Interactions Hôte-Pathogènes INP, Ecole Nationale Vétérinaire de Toulouse, 23 chemin des Capelles, BP 87614, 31076 Toulouse Cedex 03, France; 3 Gastrointestinal Laboratory, Department of Small Animal Clinical Sciences, College of Veterinary Medicine and Biomedical Sciences, Texas A&M University, College Station, TX 77843–4474, USA

**Keywords:** Dogs, Calprotectin, IgA, Age, Gestation, Biomarkers

## Abstract

Faecal calprotectin and IgA have been suggested as non-invasive markers of gut health. Faecal calprotectin is a marker of intestinal inflammation in adults, whereas IgA has been suggested as a marker of intestinal immunity. The purpose of the present study was to evaluate the effect of gestation, lactation and age on faecal concentrations of these biomarkers. Thirty puppies, nineteen pregnant or lactating bitches and eighty-nine healthy control adult dogs were included in the study. Faeces were collected from the fourth week of gestation until the eighth week of lactation in pregnant and lactating bitches, and between 4 and 9 weeks of age in puppies. Faeces from the eighty-nine healthy control adult dogs were also collected. Faecal calprotectin and IgA concentrations were measured. Faecal calprotectin concentrations in control dogs were significantly lower than faecal calprotectin concentrations in puppies between 4 and 6 weeks of age (*P* < 0·001) or between 7 and 9 weeks of age (*P* = 0·004). Puppies between 4 and 6 weeks of age had significantly higher faecal IgA concentrations compared with puppies between 7 and 9 weeks of age (*P* = 0·001). Bitches during their second month of lactation had significantly lower faecal IgA concentrations compared with their first month of lactation (*P* = 0·049). Faecal calprotectin and IgA have been suggested as non-invasive and easily measured biomarkers of gut health in adults. However, the present study underlines that faecal IgA and calprotectin concentrations vary markedly depending of physiologic factors such as gestation, lactation and age. These factors need to be considered when these faecal biomarkers are used for evaluation of intestinal immunity or inflammation.

There is increasing interest in non-invasive and minimally invasive biomarkers of disease. Validated markers may be used as an objective indicator for onset of an illness, aid in the classification of a diseased and non-diseased state, offer an evaluation of disease severity, and provide the ability to follow disease progression. Faecal biomarkers are also used to evaluate the impact of nutrition on the digestive tract in different species such as human subjects^(^^1^^)^, dogs^(^^2^^)^ or rats^(^^3^^)^.

Calprotectin, a heterodimeric protein complex mainly present in neutrophils, monocytes and reactive macrophages, and IgA have been suggested as non-invasive markers of gut health in adults. Secretory IgA is the predominant Ig subtype present in secretions, protecting mucosal surfaces of the body from infectious agents. Therefore, faecal IgA has been suggested to be the most useful marker of the mucosal immunity^(^^4^^)^. Faecal IgA deficiency in human subjects has been associated with chronic gastrointestinal disease^(^^5^^)^. In dogs, faecal secretory IgA concentrations were also used to evaluate intestinal immunity ^(^^6^^,^^7^^)^. In human subjects, faecal calprotectin concentrations were reported to be increased in patients with Crohn's disease or ulcerative colitis compared with healthy controls^(^^8^^–^^14^^)^. Moreover, a good correlation between faecal calprotectin concentrations and disease severity, as determined by endoscopy and histologic examination of biopsy samples has been demonstrated in human subjects^(^^15^^–^^17^^)^. In dogs, significantly increased serum calprotectin concentrations have been reported in patients with idiopathic inflammatory bowel disease^(^^18^^)^. Recently, faecal canine calprotectin has also been shown to increase in case of intestinal inflammation in adult dogs with chronic diarrhoea^(^^19^^)^.

In puppies, different enteropathogens, such as canine parvovirus type 2, can induce severe intestinal lesions and an immunodepression^(^^20^^)^. Evaluation of calprotectin and IgA for the management of these infectious diseases could be interesting. However, some physiological factors may impact on the concentration of these biomarkers. In human subjects, an effect of age on faecal calprotectin concentration was described with higher concentrations in healthy children compare with healthy adults^(^^21^^,^^22^^)^. In dogs, an effect of age on IgA concentrations was also described, with a lower concentration of IgA in puppies at 5 months of age compare with adult dogs^(^^23^^)^. So the purpose of the present study was to evaluate the effect of physiological factors such as gestation, lactation and age on faecal IgA and calprotectin concentrations in dogs.

## Material and methods

The study protocol was reviewed and approved by the Royal Canin Internal Ethics Committee.

### Dogs

Nineteen bitches from a French breeding kennel were followed from their fourth week of gestation until the end of their lactation (eighth week of lactation). Thirty puppies from nine litters living in the same breeding kennel were followed from 4 to 9 weeks of age. All puppies stayed with their dam in heated whelping boxes from birth to 9 weeks of age, when they were sold. Each puppy was treated with a single dose of diclazuril (Vecoxan^®^; Janssen Animal Health, 2·5 mg/kg, per os) at 4 and 7 weeks of age and with fenbendazole (Panacur^®^, 50 mg/kg, per os, q 24 h) for three consecutive days at 2, 4, 6 and 8 weeks of age. Puppies were vaccinated at 5, 6 and 7 weeks of age with a non-adjuvant, modified-live vaccine containing parvovirus Cornell 780916-115 strain with a viral titre of 10^5·5^ TCID50 (Primodog^®^). Pregnant and lactating bitches and puppies were fed the same diet, a dry expanded complete diet balanced for growing dogs (food composition: moisture 8 %, protein 30 %, crude fat 22 %, crude fibre 1·8 % and ash 6·9 %; metabolisable energy: 17 786 kJ/kg (4251 kcal/kg) (Table 1). Sixty-nine pet dogs of various breeds served as reference for faecal calprotectin. These dogs were located at the Texas A&M University (College Station, United States). Twenty other adult pet dogs served as reference for IgA. These dogs were included at Alfort National Veterinary School (Maisons-Alfort, France). Each dog was determined to be healthy based on history, physical examination findings and an evaluation of faecal quality (dogs had to have formed, but not hard faeces).

**Table 1.** Composition of diet fed to pregnant and lactating bitches and to puppies included in the study

Chicken meal, brewers rice, chicken fat, wheat gluten, maize, dried beet pulp, natural flavours, fish oil, sodium silico aluminate, vegetable oil, potassium phosphate, salt, calcium carbonate, l-lysine, fructooligosaccharides, hydrolysed yeast, choline chloride, butyrate, taurine, potassium chloride, l-tyrosine, vitamins (dl-alpha tocopherol (source of vitamin E), l-ascorbyl-2-polyphosphate (source of vitamin C), biotin, D-calcium panthotenate, vitamin A acetate, niacin, pyridoxine hydrochloride (vitamin B_6_), thiamine mononitrate (vitamin B_1_), riboflavine (vitamin B_2_), folic acid, vitamin B_12_ supplement, vitamin D_3_ supplement), marigold extract, Trace minerals (zinc proteinate, zinc oxide, ferrous sulphate, manganese proteinate, copper proteinate, copper sulphate, manganous oxide, calcium iodate, sodium selenite), l-carnitine, betacarotene, preserved with natural tocopherols, rosemary extract and citric acid.

### Faecal calprotectin and IgA assays

All faecal samples were collected just after a spontaneous defecation. In bitches from the breeding kennel, faeces were collected at three different periods of time: between the 4 and 8 weeks of gestation, between the 1 and 4 weeks of lactation and between the 4 and 8 weeks of lactation. In puppies, faeces were collected between 4 and 6 weeks of age and between 7 and 9 weeks of age. All samples (2–10 g of faeces) were then kept frozen at −20°C until analysis. One faecal sample was also collected from the eighty-nine healthy control dogs. Calprotectin and IgA were quantified by a RIA and ELISA, respectively, as previously described^(^^6^^,^^24^^)^.

### Statistical analyses

Statistical analyses were performed using Tanagra^®^ freeware (Rakotomalala, 2005). All datasets were tested for normality by the Shapiro–Wilk test. Faecal calprotectin and IgA concentrations were not normally distributed, so these data were presented as medians and ranges. A Mann–Whitney *U* test or a Kruskal–Wallis test was used for unpaired data according to the number of groups considered. As data were matched for puppies and for pregnant and lactating bitches, Wilcoxon signed-rank test or Friedman's ANOVA by rank were used depending of the number of groups. The level of statistical significance was set at *P* < 0·05 for all analyses.

## Results

### Faecal calprotectin and IgA concentrations in puppies

**Fig. 1.**
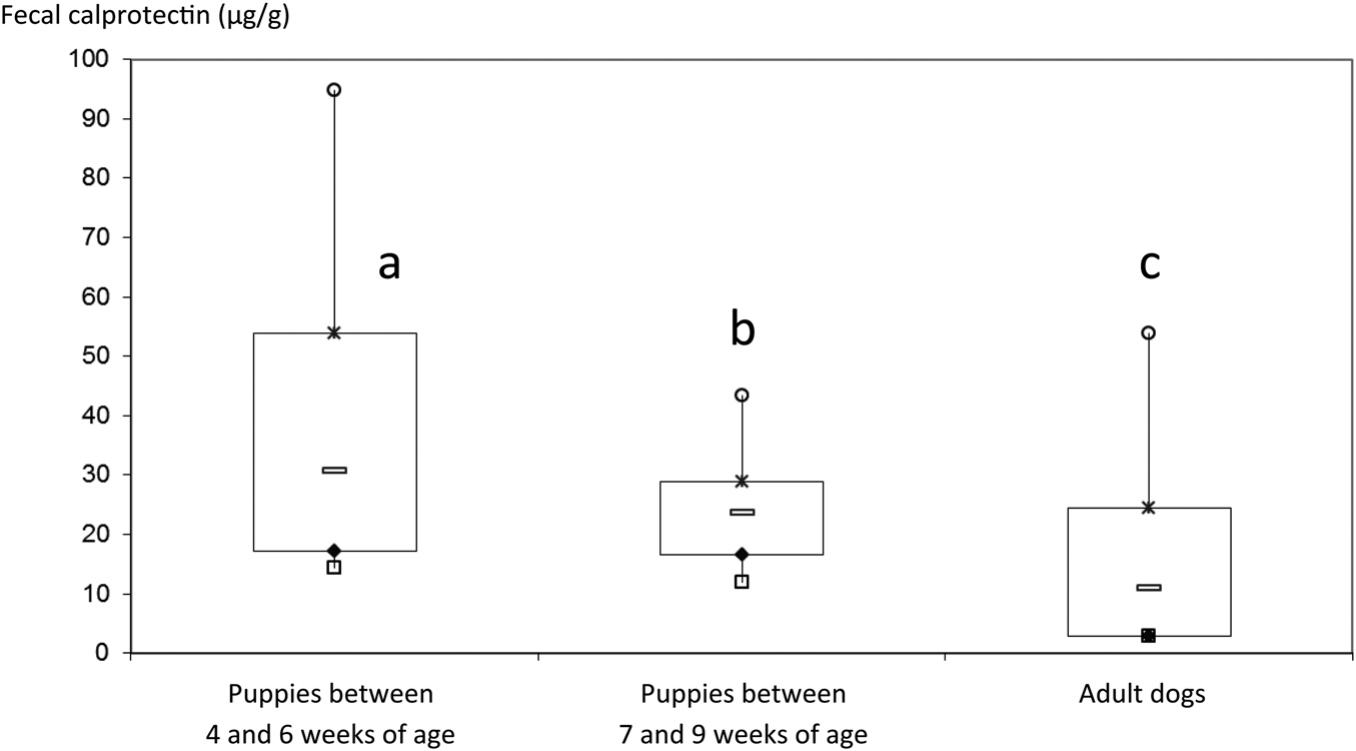
Box-and-whisker plot of faecal calprotectin concentrations in sixty-nine healthy control dogs and thirty puppies between 4 and 9 weeks of age. Each box represents the first to third quartiles (25th–75th percentiles), the bar in each box represents the median, and the whiskers represent the first to ninth decile (10th–90th percentiles).a,b,c: Values with different letters differ significantly (*P* < 0·05).

Puppies between 4 and 6 weeks of age had significantly higher faecal calprotectin concentrations compared with puppies between 7 and 9 weeks of age (30·6 (7·3–295·8) μg/g *v.* 23·6 (2·9–58·8) μg/g; *P* = 0·039). Faecal calprotectin concentrations in control dogs (11 (2·9–109·8) μg/g) were significantly lower than faecal calprotectin concentrations in puppies between 4 and 6 weeks of age (*P* < 0·001) or between 7 and 9 weeks of age (*P* = 0·004; Fig. 1).

Puppies between 4 and 6 weeks of age had significantly higher faecal IgA concentrations compared with puppies between 7 and 9 weeks of age (9·6 (0·5–29·3) mg/g *v.* 5·9 (1·2–13·1) mg/g; *P* = 0·001). Faecal IgA concentrations in adult control dogs (7·3 (0·8–21·5) mg/g) were not significantly different from faecal IgA concentrations in puppies between 4 and 6 weeks of age (*P* = 0·118) or between 7 and 9 weeks of age (*P* = 0·766).

### Faecal calprotectin and IgA concentrations in pregnant and lactating bitches

Faecal calprotectin concentrations were not significantly different during gestation (*n* 19; 3·3 (2·9–36·2) μg/g), the first month of lactation (*n* 19; 4·4 (2·9–30·3) μg/g) and the second month of lactation (*n* 19; 3·9 (2·9–24·3) μg/g; *P* = 0·985). Faecal calprotectin concentrations in control adult dogs (*n* 69; 11 (2·9–109·8) μg/g) tended to be higher than faecal calprotectin concentrations in bitches during pregnancy (*P* = 0·08) and during their second month of lactation (*P* = 0·055).

Bitches during their second month of lactation had significantly lower faecal IgA concentrations (*n* 19; 2·7 (0·4–19·6) mg/g) compared with their first month of lactation (*n* 19; 7 (0·4–19·6) mg/g; *P* = 0·049) and tended to have lower concentrations than during their pregnancy (*n* 19; 9·2 (0·8–18·4) mg/g; *P* = 0·07). Faecal IgA concentrations in control adult dogs (*n* 20; 7·3 (0·8–21·5) mg/g) were not significantly different from faecal IgA concentrations in bitches during pregnancy (*P* = 0·673) and during their first month of lactation (*P* = 0·736); and tended to be higher than concentrations in bitches during their second month of lactation (*P* = 0·092).

## Discussion

Faecal calprotectin concentrations showed a significant association with age in puppies, with higher faecal concentrations observed in puppies between 4 and 6 weeks of age compared with those puppies between 7 and 9 weeks of age. This result is in accordance with studies in human subjects in which higher faecal calprotectin concentrations were observed in infants in their first year compared with healthy adolescents or adults^(^^1^^,^^21^^,^^25^^)^. These high faecal calprotectin concentrations in young puppies are similar to those of adult dogs with inflammatory intestinal pathology^(^^19^^)^. However, these high concentrations could be explained by other factors than an intestinal inflammation. The type of food (e.g., natural milk, industrial milk and dry food) could influence faecal calprotectin concentrations. In human subjects, infants exclusively breastfed show significantly higher faecal calprotectin concentrations compared with those who receive a mixed diet^(^^1^^,^^22^^)^. This could be due to hormones (such as ghrelin and leptin), cytokines and other immunostimulating and growth factors (such as epidermal growth factor and granulocyte colony-stimulating factor) in human milk, which contribute to the development of the gastrointestinal immune system^(^^22^^)^. Developmental processes occurring in the digestive tract during this stage of life could also explain these higher faecal calprotectin concentrations. During the first weeks of life puppies have increased intestinal permeability^(^^26^^)^, which may lead to transepithelial migration of neutrophils, as observed in adults with IBD^(^^27^^)^. The physiological establishment of the gut microbiota may also have an effect on calprotectin release as has been suggested in human subjects^(^^28^^,^^29^^)^. As calprotectin has many biological activities, including bactericidal and fungicidal properties, it may be postulated that this protein may have a protective role for the immature neonatal intestines, until the epithelial tight junction proteins and other factors of intestinal immunity develop with age^(^^30^^)^.

Puppies between 4 and 9 weeks of age did not show significant differences in faecal IgA concentrations compared with adult dogs. This result differs from a previous study in which significantly lower faecal IgA concentrations were described in puppies at 5 months of age compared with adult dogs^(^^23^^)^. This lack of difference in the present study could be explained by the different origins of dogs and so the different environmental conditions (puppies living in breeding kennel *v.* adult pet dogs). This high concentration of IgA observed in the present study could also be due to the consumption of milk by puppies before 9 weeks of age. The nature of the placenta in the dog prevents transfer of Ig from the maternal to the fetal circulation. Hence, the newborn dog is dependent on antibodies and other factors from colostrum and milk for disease resistance. During the first 24 h of life colostrum provides mainly IgG, which is absorbed into the vascular space^(^^31^^)^. During lactation, IgG concentrations decrease and IgA concentrations increase to become the main Ig fraction in milk^(^^32^^)^. Thus, the ingestion of milk could contribute to high faecal IgA concentrations in these puppies. In human subjects, exclusively breast-fed children show higher faecal IgA concentrations than exclusively formula-fed infants, with a high correlation between secretory IgA intake and output^(^^33^^–^^35^^)^. The significant decrease in faecal IgA concentrations in puppies between 7 and 9 weeks of age compared with puppies between 4 and 6 weeks of age may be due to a decrease in milk consumption.

Faecal IgA concentrations were lower in bitches during their second month of lactation compared with their first month of lactation. This may be explained by the important production of IgA in the milk and the high quantity of milk produced by bitches during this period. This high mobilisation of IgA in milk could induce a decrease of faecal IgA. This low faecal IgA concentration could underline an immunity gap during this period, which could predispose bitches to excrete enteropathogens (*Cystoisospora* sp., canine parvovirus type 2, *Giardia* sp.).

### 

#### Conclusion

Faecal calprotectin and IgA have been suggested as non-invasive and easily measured biomarkers of gut health in adults. However, the present study underlines that faecal IgA and calprotectin concentrations vary markedly depending of physiologic factors such as gestation, lactation and age. These factors need to be considered when these faecal biomarkers are used for evaluation of intestinal immunity or inflammation.
